# Did We Overreact? Insights on COVID-19 Disease and Vaccination in a Large Cohort of Immune-Mediated Inflammatory Disease Patients during Sequential Phases of the Pandemic (The BELCOMID Study)

**DOI:** 10.3390/vaccines12101157

**Published:** 2024-10-11

**Authors:** Jeroen Geldof, Marie Truyens, João Sabino, Marc Ferrante, Jo Lambert, Hilde Lapeere, Tom Hillary, An Van Laethem, Kurt de Vlam, Patrick Verschueren, Triana Lobaton, Elizaveta Padalko, Séverine Vermeire

**Affiliations:** 1 Department of Gastroenterology and Hepatology, Ghent University Hospital, 9000 Ghent, Belgium; marie.truyens@uzgent.be (M.T.); triana.lobatonortega@uzgent.be (T.L.); 2 Department of Internal Medicine and Pediatrics, Ghent University, 9000 Ghent, Belgium; 3 Department of Gastroenterology and Hepatology, University Hospitals Leuven, 3000 Leuven, Belgium; joao.sabino@uzleuven.be (J.S.); marc.ferrante@uzleuven.be (M.F.); severine.vermeire@uzleuven.be (S.V.); 4 Translational Research in Gastrointestinal Disorders (TARGID), Department of Chronic Diseases and Metabolism (CHROMETA), KU Leuven, 3000 Leuven, Belgium; 5 Department of Dermatology, Ghent University Hospital, 9000 Ghent, Belgium; jo.lambert@uzgent.be (J.L.); hilde.lapeere@uzgent.be (H.L.); 6 Department of Dermatology, University Hospitals Leuven, 3000 Leuven, Belgium; tom.hillary@uzleuven.be (T.H.); an.vanlaethem@uzleuven.be (A.V.L.); 7 Department of Rheumatology, University Hospitals Leuven, 3000 Leuven, Belgium; kurt.devlam@uzleuven.be (K.d.V.); patrick.verschueren@uzleuven.be (P.V.); 8 Department of Laboratory Medicine, Ghent University Hospital, 9000 Ghent, Belgium; elizaveta.padalko@uzgent.be

**Keywords:** IMID, COVID-19, vaccination, booster, real world

## Abstract

Introduction: As the COVID-19 pandemic becomes an endemic state, still many questions remain regarding the risks and impact of SARS-CoV-2 infection and vaccination in patients with immune-mediated inflammatory diseases (IMIDs) who were excluded from the phase 3 COVID-19 vaccination trials. Methods: The BELCOMID study collected patient data and serological samples from a large, multicentric IMID patient cohort that was prospectively followed during sequential stages of the pandemic. Patients were stratified according to vaccination status into five groups across three sampling periods. Interactions between SARS-CoV-2 infection, COVID-19 vaccination status, IMID-treatment modalities and IMID course were explored. Results: In total, 2165 patients with IBD, a dermatological or rheumatological IMID participated. SARS-CoV-2 infection rates increased over the course of the pandemic and were highest in IMID patients that had refused every vaccine. After baseline COVID-19 vaccination, serologic spike (S)-antibody responses were attenuated by particular types of immune-modulating treatment: anti-TNF, rituximab, JAKi, systemic steroids, combined biologic/immunomodulator treatment. Nonetheless, S-antibody concentration increased progressively in patients who received a booster vaccination, reaching 100% seroconversion rate in patients who had received two booster vaccines. Previous SARS-CoV-2 infection was found as a predictor of higher S-antibody response. Patients who had refused every vaccine showed the lowest rates of S-seroconversion (53.8%). Multiple logistic regression did not identify previous SARS-CoV-2 infection as a risk factor for IMID flare-up. Furthermore, no increased risk of IMID flare-up was found with booster vaccination. Conclusions: Altogether, the BELCOMID study provides evidence for the efficacy and safety of COVID-19 vaccination and confirms the importance of repeated booster vaccination in IMID patients.

## 1. Introduction

The SARS-CoV-2 pandemic and subsequent vaccination campaign is considered a milestone in recent medical history and has led to several structural changes in medical care worldwide. In chronic immune-mediated inflammatory disease (IMID) care in particular, the pandemic has triggered remarkable evolutions.

IMIDs such as inflammatory bowel disease (IBD), rheumatologic arthropathies and immune-mediated skin diseases are believed to originate from an inappropriate immune response to environmental triggers in genetically susceptible hosts. They have an estimated prevalence of 5–7% in developed countries and a rapid rise in incidence is being observed in developing countries in Asia, South-America and in the Middle-East [1,2,3]. Over the past two decades, medical treatment options for IMIDs expanded substantially with the arrival of anti-cytokine therapies targeting tumor necrosis factor (TNF) alpha, interleukin (IL)12/23, IL17, IL6, T- and B-cell targeting therapies and small molecules such as Janus kinase inhibitors (JAKi), sphingosine-1-phosphate receptor modulators and the PDE4 inhibitor apremilast. The risk of infections has been generally considered higher in patients under these targeted immune-modulating therapies (TIMT), which was a serious concern for increased SARS-CoV-2 infection risk and more severe COVID-19 disease (defined as COVID-19 disease leading to hospitalization, intensive care unit admission, ventilation and/or mortality) amongst IMID patients and their caregivers in the early days of the pandemic. This resulted in more stringent shielding advice [4,5] and also provoked a sudden shift from inpatient care to increased outpatient care, use of telemedicine and faster uptake of subcutaneous formulations of biologic treatment modalities instead of their intravenous forms [6]. Furthermore, upon initiation of national COVID-19 vaccination campaigns, IMID patients were prioritized for baseline and (repeated) booster vaccinations.

Fast forward 4 years later, COVID-19 and SARS-CoV-2 are far less trending subjects. COVID-19 has grown into an endemic disease with recurrent (smaller) peaks. Still many questions remain such as the impact of SARS-CoV-2 infection and COVID-19 vaccination on the IMID disease course, the potential influence of immunosuppressive treatment modalities on vaccine efficacy, and the presumed need for further booster vaccination in IMID patients in the long run.

The BELCOMID study explored the interactions between SARS-CoV-2 infection, COVID-19 vaccination status, IMID disease course and immune-modulating treatment modalities in a large, multicentric IMID patient cohort that was prospectively followed during different stages of the pandemic. Our study aimed to provide real-world evidence as to whether IMID patients are at increased risk of (severe) COVID-19 disease; to whether immunological responses to COVID-19 disease and vaccination are influenced by IMID diseases or by their particular treatment modalities; and to whether COVID-19 infection or vaccination may induce changes in IMID disease activity.

Interim results of the study were published previously and suggested a rather benign course of COVID-19 disease in IMID patients prior to vaccination as well as a blunting effect from systemic steroids, TIMT and/or immunomodulator treatment on serologic COVID-19 vaccination responses after baseline vaccination [7]. The current manuscript adds data on COVID-19 booster vaccinations, focusses further on the different COVID-19 vaccination statuses (described below) within the IMID patient cohort and places the results in perspective within the available IMID literature with the goal of providing a comprehensive summary on what healthcare professionals should keep focus on during continued care for IMID patients in the ongoing SARS-CoV-2 pandemic/endemic.

## 2. Materials and Methods

### 2.1. Study Population and Design

In March 2020, at the beginning of the SARS-CoV-2 pandemic, an interdisciplinary consortium was constructed between the University Hospital, Leuven and the Ghent University Hospital (Belgium). A prospective, observational cohort study was developed within this consortium to monitor the course of the pandemic in a large cohort of IMID patients, the BELCOMID study. The study was approved by the ethics committees of both university hospitals (BC-08030/S64422).

Consecutive patients with gastrointestinal, rheumatological or dermatological IMIDs (Crohn’s disease, ulcerative colitis, spondylarthritis, psoriatic arthritis, rheumatoid arthritis, psoriasis, hidradenitis suppurativa or atopic dermatitis) followed at one of either tertiary centers, were invited to participate between 17 December 2020 and 28 February 2021. IMID patients were eligible for study inclusion regardless of their current treatment modality. Conventional IMID treatment options included therapies without immunomodulatory effect (N-IM) and immunomodulators (IMM). N-IM comprised sulfasalazine, mesalazine, acitretin, metformin, zinc, antibiotics, topical treatment options or light therapy. TIMT options included all available biologics and small molecules at that moment in time.

**Figure 1:**
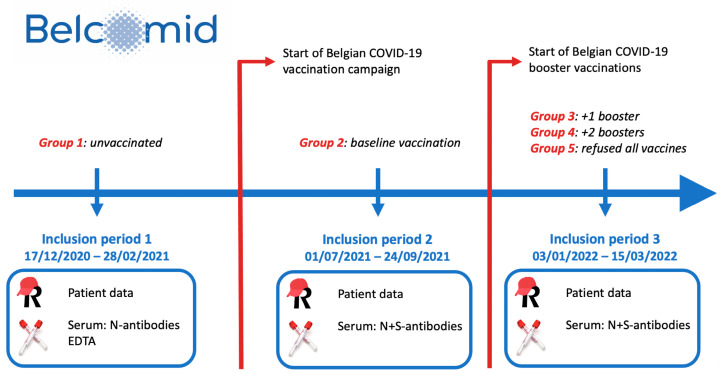
BELCOMID timeline. Interim results from inclusion periods 1 and 2 were published previously [7]. The current manuscript considers all 3 inclusion periods and 5 vaccination groups.

Participating patients were followed prospectively. Patient data were provided by patients themselves through questionnaires and completed with data from the electronic patient file by their treating physician. Serial blood samples were drawn. Both patient data and blood samples were collected at three predefined inclusion periods. These periods were carefully selected based on the evolution of the pandemic and governmental vaccination strategy from December 2020 to February 2022 with a minimum time interval of 4 months in between sequential sampling (see Figure 1). At sampling period 1, participants were evaluated prior to the start of the national COVID-19 vaccination campaign. Sampling period 2 evaluated patients before the start of booster vaccinations and sampling period 3 after the start of the booster vaccination campaign.

The goal of the BELCOMID study was threefold. The initial aim was to use this large, real-life IMID patient cohort to explore the association between COVID-19 and IMIDs. This involved prospective analysis of exposure to and infection with SARS-CoV-2 and relating this information to the underlying IMID disease course and respective treatment modality. As the pandemic progressed and national vaccination campaigns kicked off, the second goal was to study the response to COVID-19 vaccination in these patients and explore factors associated with serological responses. For this purpose, 5 different patient groups were identified within the BELCOMID cohort across the 3 sampling periods according to their vaccination status (Figure 1). Group 1: patients without COVID-19 vaccination evaluated before onset of the national vaccination campaign (sampling period 1). Group 2: patients evaluated after onset of the national vaccination campaign who received complete baseline (2 doses of mRNA-1273, BNT162b2, ChadOx1 nCoV-19 or 1 dose of JN78436735) COVID-19 vaccination (sampling period 2). Group 3: patients evaluated after start of booster vaccinations who had received 1 extra booster vaccine (sampling period 3). Group 4: patients who had received 2 booster vaccines (sampling period 3), Group 5: patients evaluated after start of the booster campaign but who had refused every vaccine so far (sampling period 3).

Thirdly, we explored the potential association between previous SARS-CoV-2 infection, COVID-19 vaccination status and IMID disease activity.

### 2.2. SARS-CoV-2 Serologic Testing

For detection of anti-nucleocapsid antibodies (N-antibodies), the Abbott Architect™ (Lake Forest, IL, USA) SARS-CoV-2 immunoglobulin G (IgG) assay (>1.4 = positive) was used. For detection of anti-spike protein antibodies (S-antibodies) the Abbott Architect™ (Lake Forest, IL, USA) SARS-CoV-2 IgGII Quant assay (≥50 AU/mL = seroconversion) was used [8,9].

In the first sampling period (Group 1), before onset of the national vaccination campaign, blood samples were analyzed for SARS-CoV-2 nucleocapsid protein (N-) antibodies to identify previous infection. COVID-19 vaccines induce a selective increase in spike protein (S-) antibodies and not N-antibodies. Therefore, at the two following evaluation timepoints (all other vaccination groups), both N- and S-antibodies were assessed to discriminate between previous infection and vaccination.

### 2.3. Endpoints

Primary endpoints in the first phase of the study were positive SARS-CoV-2 PCR test (nasopharyngeal swab) and SARS-CoV-2 serology reflecting SARS-CoV-2 infection or vaccination. During the second phase of the study in each vaccination group, potential associations were explored between infection and vaccination with IMID-treatment modality, IMID-disease activity (using validated disease activity scores), IMID type, smoking status, increased SARS-CoV-2 exposure risk and previous SARS-CoV-2 infection. Last but not least, associations between SARS-CoV-2 infection or vaccination and IMID flare-up were explored.

### 2.4. Data Collection and Statistical Analyses

The REDCap^®^ (Vanderbilt University, Nashville, TN, USA) electronic case report form was used to collect patient and serologic data pseudonymously. IBM SPSS Statistics (for Windows, Version 29.0.2.0 Armonk, NY, USA) was used for descriptive statistics. The Ghent University Biostatistics unit performed all exploratory analyses in R version 4.0.2 (University of Auckland, New Zealand).

Both marginal and conditional associations were tested. For marginal associations, two-sided Pearson’s chi-squared tests were used. Conditional effects were tested using binary logistic regression models adjusted for the propensity score of the respective treatment and multiple logistic regression analyses. The propensity score was estimated by fitting a logistic regression model where treatment was the response and potential confounders were the predictors. Potential confounders included age category, gender, smoking status, SARS-CoV-2 exposure risk, BMI category, comorbidities, and previous SARS-CoV-2 infection.

The continuous serology outcome (concentration) was log-transformed (log(mu + 0.1)) to allow linear regression analysis.

All hypothesis testing was performed at the 0.05 significance level. Confidence intervals for risk ratios were calculated using normal approximation (Wald test statistic). No adjustment for multiple testing was made as the analyses are considered to be exploratory and hypothesis generating. Therefore, results should be interpreted with caution and require confirmation by other research.

## 3. Results

The results of the first and second sampling periods were published previously [7]. The current manuscript adds the results of the third sampling period and focusses on the results per vaccination status described according to the previously defined five vaccination groups (see above).

### 3.1. Demographics Per Vaccination Group

At baseline, 2165 patients participated. Of these, 1566 proceeded to participate during all three sampling periods. Demographics for each of the five predefined vaccination status groups are shown in Table 1. Apart from Group 5, all groups had comparable demographics with a balanced sex distribution. Over the three inclusion periods, the distribution of immune-modulating treatment modalities for IMID disease was relatively stable.

Group 5, a small group of patients who refused every vaccination up to the third sampling timepoint, were generally younger (mean age of 39.4 years, SD 14.4—age < 60 y: 87.5%, *p* < 0.001), had the lowest BMI (mean 23.8, SD 3.363—BMI > 25: 35%, *p* < 0.001), the highest numerical rate of active smokers (30%, *p* < 0.001) and the largest number of patients treated with TIMT (95%, *p* < 0.001). Group 5 contained numerically more male patients (60%, *p* = 0.738).

### 3.2. PCR Positivity Rate and Serologic Analyses per Vaccination Group

Results for PCR positivity rates, nucleocapsid and spike antibody seroconversion rates per vaccination group are shown in Table 2. Prior to the vaccination campaign, at the first inclusion period (Group 1), 5.1% of all participants had a positive SARS-CoV-2 PCR. At the third sampling timepoint, over a fifth of all vaccinated patients (Group 3: 20.5%, Group 4: 28.6%) had a previous positive SARS-CoV-2 PCR. As expected, these results were mimicked by the N-seropositivity rates that increased over time and were higher in the vaccination groups from the third inclusion period. However, the rates of previous SARS-CoV-2 infection were significantly higher in patients who had refused every vaccine up to the third inclusion period (Group 5: 50%, *p* < 0.001).

**Figure 2:**
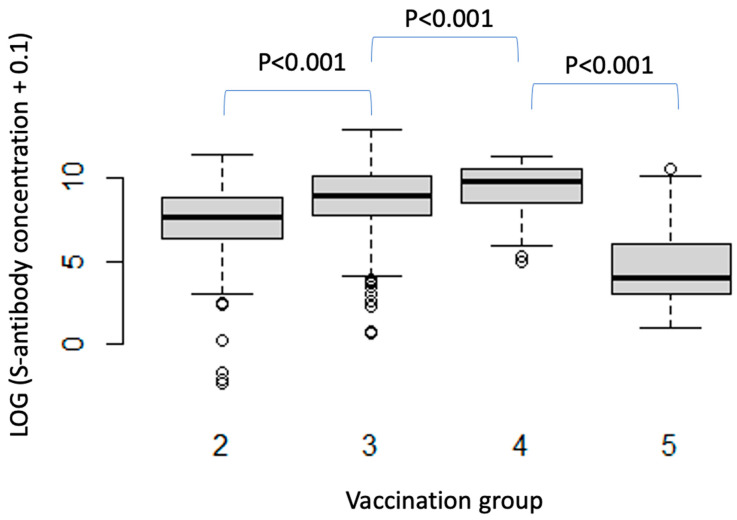
Estimated mean S-serology evolution across vaccination groups.

This observation was mirrored across the vaccination groups. S-antibody seroconversion rate was 95.1% after baseline vaccination (Group 2), increased to 98% after one booster vaccine (Group 3) and was 100% in patients having received two booster vaccines (Group 4). Furthermore, repeated vaccination led to a progressive increase in mean S-concentration (Figure 2). The estimated mean S-serology concentration was 3.67 times higher for patients who received one booster vaccine (Group 3) compared to patients who had received baseline vaccination only (Group 2) (95% CI 3.27–4.12, *p* < 0.001). Additional booster vaccination (Group 4) led to a further 79% increase in estimated geometric mean S-antibody concentration compared to single booster vaccination (Group 3) (95% CI 1.34–2.39, *p* < 0.001).

Vice versa, in Group 5, 53.8% of patients who had refused vaccination did not show S-antibody seroconversion and 41% neither had S- nor N-antibody seroconversion.

### 3.3. Impact of IMID-Treatment Modality on SARS-CoV-2 and Vaccination Response

A summary of all analyses exploring associations between IMID-treatment modality and SARS-CoV-2 response (PCR testing and serologic results) per vaccination group can be found in Table 3.

The results of SARS-CoV-2 PCR and N-serology analysis showed no clear associations with TIMT, IMM or the use of systemic steroids in IMID patients before vaccination (Group 1). However, looking into subgroups of TIMT, a higher odds of PCR positivity (OR 2.51, 95% CI 0.95–5.95, *p* = 0.047) and N-seroconversion (OR 4.54, 95% CI 1.60–11.10, *p* < 0.01) was found in IMID patients treated with IL23 inhibitors in Group 1. In the same group, anti-TNF, by contrast, was associated with significantly reduced odds of N-seroconversion compared to anti-IL12/23/17 treatment (OR 0.39, 95% CI 0.18–0.88, *p* = 0.02) and vedolizumab treatment (OR 0.40, 95% CI 0.18–0.86, *p* = 0.019). After onset of the national vaccination campaign, all differences between treatments disappeared with repeated vaccination.

With regard to S-antibody response after baseline vaccination (Group 2), the odds of S-antibody seroconversion were significantly lower with TIMT (OR 0.28, 95% CI 0.10–0.65, *p* < 0.01), IMM (OR 0.28, 95% CI 0.16–0.49, *p* < 0.001), combined TIMT/IMM (OR 0.16, 95% CI 0.09–0.28, *p* < 0.001) and systemic steroid treatment (OR 0.18, 95% CI 0.10–0.37, *p* < 0.001). Within the TIMT-treated patient group, patients treated with rituximab had significantly lower odds of S-seroconversion (OR 0.04, 95% CI 0.01–0.10, *p* < 0.001). Patients treated with anti-TNFs had a significantly lower mean S-antibody concentration (mean ratio 0.57, 95% CI 0.03–0.16, *p* < 0.01) and patients treated with JAK-inhibitors significantly higher odds of being in the lowest quartile of S-antibody concentrations (OR 2.48, 95% CI 1.12–5.24, *p* = 0.019). The S-antibody seroconversion rate and mean S-antibody concentrations of anti-TNF alpha-treated patients were still significantly higher than the concentrations of rituximab-treated patients (respectively: OR 26.3, 95% CI 7.36–105, *p* < 0.001—mean ratio 11.1, 95% CI 11.1, 95% CI 4.18–29.4, *p* < 0.001). Comparable observations were made in the patient group who received one booster vaccine (Group 3).

Vice versa, in Group 2, treatment with vedolizumab and anti-IL23 was associated with significantly higher S-antibody concentrations (respectively: mean ratio 1.84, 95% CI 1.40–2.41, *p* < 0.001—mean ratio 2.17, 95% CI 1.19–3.96, *p* = 0.011). However, these treatment modalities were not found to have a significant impact on S-antibody seroconversion rates. This observation persisted in patients who received one booster vaccine (Group 3), and in this group, patients treated with vedolizumab or anti-IL12/23 had significantly even lower odds of being in the lowest S-antibody quartile (respectively: OR 0.21, 95% CI 0.12–0.34, *p* < 0.001—OR 0.47, 95% CI 0.23–0.87, *p* = 0.023).

As previously mentioned, patients who had received two booster vaccines showed 100% S-antibody seroconversion rate.

In Group 5, which consisted of a relatively small number of patients, no significant associations between IMID-treatment modality and SARS-CoV-2 PCR or serologic response could be identified.

### 3.4. Other Influencing Factors

Multiple logistic regression analysis revealed that previous SARS-CoV-2 infection was a predictor of higher S-antibody response in patients in Group 2 and Group 3 (lower odds of lowest S-antibody quartile, respectively: OR 0.44, 95% CI 0.21–0.78, *p* < 0.01–OR: 0.17, 95% CI 0.10–0.27, *p* < 0.001).

In patients who received one booster vaccine (Group 3), multiple logistic regression analysis revealed age category to be a significant predictor for PCR positivity and N-seroconversion (≥60 yo, respectively: OR 0.45, 95% CI 0.28–0.71, *p* < 0.001–OR 0.42, 95% CI 0.26–0.65, *p* < 0.001).

Active smoking was associated with lower S-antibody response after baseline (risk of lowest S-antibody quartile—Group 2: RR 1.45, 95% CI 1.11–1.89, *p* < 0.01) and after one booster vaccination (risk of lowest S-antibody quartile—Group 3: RR 1.42, 95% CI 1.10–1.84, *p* = 0.0122). However, in these groups, smoking did not influence S-antibody seroconversion rates. Furthermore, after two booster vaccines (Group 4), this association was no longer significant (RR 0.46, 95% CI 0.11–1.82, *p* = 0.3752).

Influence of IMID type was explored with regard to vaccination response. Both in Group 2 and Group 3, rheumatological-IMID patients were found to have a significantly higher risk of being in lowest quartile of S-antibody concentration compared to dermatological-IMID (Group 2 derm vs. rheum: RR 0.45, 95% CI 0.30–0.66, *p* = 0.001—Group 3 derm vs. rheum: RR 0.51, 95% CI 0.34–0.77, *p* = 0.001) and IBD patients (Group 2 rheum vs. IBD: RR 1.7, 95% CI 1.30–2.10, *p* < 0.001—Group 3 rheum vs. IBD: 1.7, 95% CI 1.40–2.20, *p* < 0.001). After additional booster vaccination (Group 4), significant differences in serological response between IMID types were no longer observed.

### 3.5. IMID Flare-Ups during the Pandemic

At the third inclusion timepoint, 439 IMID patients (25.17%) had reported an IMID flare-up. Of these, 28.47% had experienced a previous SARS-CoV-2 infection but no association between IMID flare-up rate and previous SARS-CoV-2 infection was found.

Prior IMID flare-up was associated with a lower risk for PCR positivity (RR 0.42, 95% CI 0.21–0.85, *p* = 0.0163) and higher odds of S-seroconversion after baseline vaccination (Group 2—OR 3.16, 95% CI 1.88–5.36, *p* < 0.001). This association was no longer observed in patients who received booster vaccinations.

There was also no increased risk of IMID flare-up with additional booster vaccination. Patients who received two booster vaccines even showed a lower RR for IMID flare-up compared to those who only received one booster (RR 0.70, 95% CI 0.56–0.89 *p* < 0.01).

## 4. Discussion

According to the WHO registry, COVID-19 disease has caused over seven million deaths worldwide as of September 2024 [10]. SARS-CoV-2 shares similarities with IMIDs in its pathogenesis which has led to concern for worse outcomes in IMID patients. In hindsight, three main topics of concern dominated IMID-research during the pandemic: (1) Are IMID patients at increased risk of COVID-19 compared to healthy individuals? (2) Is vaccine efficacy reduced in IMID patients? (3) Can SARS-CoV-2 infection and/or COVID-19 vaccination trigger flares of the underlying IMID? The results of the BELCOMID study provide insights into each of these three.

Prior to the onset of the national vaccination campaign, we found low rates of SARS-CoV-2 infection (Group 1) (PCR positivity 5.1%, N-seroconversion 3.1%). However, it remains debatable whether the infection risk in unvaccinated IMID patients is actually significantly different from that of unvaccinated healthy controls. On the one hand, a systematic review including >300,000 patients and meta-analysis of seven case-controlled studies until July 2020, found that the risk of COVID-19 in autoimmune disease patients was significantly higher compared to healthy controls (OR 2.19, *p* = 0.038) but without increased risk of severe COVID-19 [11]. On the other hand, later published, large, observational cohort studies did not show increased COVID-19 rates in IMID patients, although the rate of COVID-19 testing [12,13,14,15], the rates of COVID-19-related hospitalizations [15,16] and the COVID-19 symptom duration [17] tended to be increased in IMID patients compared to healthy controls.

Theoretically, immune-modulating treatment modalities could interfere with the potential SARS-CoV-2 cytokine storm leading to a milder COVID-19 disease course in unvaccinated IMID patients [14,18,19]. Considering serological immune response following SARS-CoV-2 infection, our results showed no clear association with TIMT in general but did suggest an attenuating effect on N-antibody response from anti-TNF treatment in Group 1. These findings are in line with other studies that did not reveal increased risk of severe COVID-19 in patients treated with biologics [11,13,14,20,21,22,23] and that specifically identified anti-TNF monotherapy as a rather protective drug against severe COVID-19 [11,24,25]. Furthermore, we did not find a significant association between the B-cell depleting agent rituximab and SARS-CoV-2 infection rate in unvaccinated patients. Although concerns were raised in the rheumatological community [26,27], this is similar to the findings of the largest rituximab-treated unvaccinated cohort of 1895 multiple sclerosis patients showing no difference in infection or mortality rate compared to healthy controls [28].

Lastly, our results show no association between corticosteroid treatment and SARS-CoV-2 infection rate prior to the start of vaccination campaign. This stands in contrast to several large-scale observational studies including the GRA-registry, the Surveillance Epidemiology of Coronavirus Under Research Exclusion for Inflammatory Bowel Disease (SECURE-IBD) registry, the dermatological PsoProtect registry as well as the systematic meta-analysis by Akiyama et al. [11,21,23,29] that all suggest increased risk of severe COVID-19. However, it remains uncertain to what extend this association might reflect causality or may be attributed to less well-controlled underlying IMID disease [30].

All available COVID-19 vaccines are non-live vaccines and so, at least theoretically, not contraindicated in patients treated with immunosuppressive or immune-modulating medical treatment. Nonetheless, patients receiving these treatments were excluded from the original trials for SARS-CoV-2 vaccine efficacy [31,32,33]. Therefore, all evidence and guidelines on vaccination in IMID patients is based on real-world data.

Considering serological response to COVID-19 vaccination, IMIDs have been associated with reduced efficacy compared to healthy controls [34]. In our study, the S-antibody seroconversion rate after baseline vaccination (Group 2) was 95.1%. This is slightly higher compared to the results of two meta-analyses that reported pooled seroresponse rates of 83% [20] and pooled seroconversion rates of 88% among IMID patients after baseline vaccination [35]. A potential explanation for the higher rate in our study might be related to SARS-CoV-2 infections previous to vaccination. Based on N-seroconversion and/or positive PCR, 8.6% of Group 2 patients already had experienced SARS-CoV-2 infection and this was found to be a significant predictor of higher S-antibody concentration. Similarly, results of the CLARITY-IBD and VIP study showed that the combination of previous SARS-CoV-2 infection and vaccination were independently associated with higher antibody titers compared to vaccination alone [36,37]. This synergistic effect has also been observed in the general population [38,39].

Antibody titers have been shown to decrease progressively over time after baseline vaccination. This decline appears as soon as 3–4 months after administration of the second vaccine dose [1,13,35,40,41] and seems more pronounced in IMID patients compared to healthy controls [42,43]. In our study, we found that in Group 2 the odds of S-antibody seroconversion were significantly lower in patients treated with TIMT, IMM, combined IMM/TIMT and systemic steroid treatment. Within the TIMT cohort, anti-TNF, JAKi and particularly rituximab (even when compared to anti-TNF) had the strongest attenuating effect. These findings are in line with other observational studies [20,35,36,41,43,44,45,46,47,48,49,50] and suggest that probably the type of immunosuppressive treatment is the primary contributor to a reduced immune response, instead of the underlying IMID. Likewise, a waning of antibody titers was shown to be more pronounced in IMID patients treated with anti-TNF [13,42,51,52] but not with anti-integrins, anti-IL17 or anti-IL12/23 [13,44,45,50].

This attenuating effect of immune-modulating treatment modalities and the increased waning of humoral responses inevitably led to concern for higher rates of breakthrough infections in IMID patients. The appearance of variants of concern (VOC) such as the Delta and Omicron strains added to this risk since the neutralizing antibody responses of vaccination appeared to be lower in comparison to wild-type SARS-CoV-2 [1,40,53,54]. However, we found a progressive increase in mean S-antibody concentration from baseline vaccination (Group 2) over booster vaccination (Group 3) to additional booster vaccination (Group 4). All patients who received two booster vaccines (Group 4) achieved S-antibody seroconversion. Similar observational cohort studies in IMID patients confirmed that booster vaccination led to increased antibody levels, elicited seroconversion in previous non-responders and even may increase the duration of the serologic response [35,49,53,55]. Furthermore, booster vaccination broadened antibody responses against variants of VOCs [46]. Therefore, (repeated) booster vaccination should be considered the golden standard to improve and extend immune responses in IMID patients treated with immune-modulating therapies and the testing of antibody concentrations may be considered to stratify the need for and timing of additional booster vaccines in IMID patients.

Immune-mediated phenomena have been described after SARS-CoV-2 infection and vaccination such as anti-phospholipid syndrome, Guillain-Barré syndrome, immune-mediated thrombocytopenia as a consequence of COVID-19 disease [35,56,57] and thrombocytopenia associated with central venous sinus thrombosis after ChAdOx1 adenoviral vector vaccination [58] or auto-immune liver disease and IgA nephropathy following mRNA-based SARS-CoV-2 vaccination [59]. Therefore, concerns have been raised about potential triggering of the underlying IMID disease secondary to SARS-CoV-2 infection and/or vaccination. In our study, over a quarter of patients had experienced IMID flare-up prior to the last sampling period. Multiple logistic regression did not identify previous SARS-CoV-2 infection as a risk factor for IMID-flare-up. Furthermore, we found no increased risk of IMID flare-up with additional booster vaccination. Other studies evaluating the secondary risk of IMID flare-up reported relatively low rates of flare-up (after vaccination: range 2–7%) [57,60,61,62,63] and these rather mild flare-ups unfrequently required treatment changes [25,62]. Still, it remains difficult to provide true estimates of the risk of increased IMID disease activity, nor is there definite proof of a causal relationship, especially in IMIDs that are often characterized by a fluctuating disease course. Altogether, the benefits of vaccination against COVID-19 are still considered to outweigh the potential risk of IMID flare-up.

The BELCOMID study has some limitations. Our study focused on serological immune response to COVID-19 vaccination and did not evaluate cellular immune responses. However, previous studies have shown good correlation between specific T-cell responses and antibody responses [52,64]. Both serological and cellular responses to COVID-19 baseline vaccination are decreased in IMID patients compared to healthy controls [1,40,46,65]. Similar to humoral responses, the impairment of cellular immune responses may be related to the different types of immune-modulating treatment modalities. JAKi tofacitinib reduced T-cell responses compared to healthy controls and T-cell responses seemed to be impaired in methotrexate-treated patients [52,54]. By contrast, although rituximab and anti-TNF treatment were associated with the most pronounced attenuation of serological responses, these treatment modalities did not interfere with reported T-cell responses [36,46,54,65].

Secondly, there has been debate about whether antibody seroconversion is a proxy indicator of protection from SARS-CoV-2 infections and vaccine-induced immune responses are often measured as correlate of vaccine efficacy. In this view, functional assays for the detection of neutralizing antibodies have been considered as surrogates of infection. In our study, we evaluated anti-S-antibody concentrations and seroconversion rates but not neutralizing antibodies. Nonetheless, several studies have shown that S-antibodies are well correlated with neutralizing antibodies [35,39,43,66,67]. Regarding vaccine efficacy, several observational studies have confirmed that COVID-19 vaccination in IMID patients is associated with reduced SARS-CoV-2 infection rates, reduced severity of COVID-19 disease and reduced symptom longevity [15,17,68,69,70]. This is in line with our study findings, showing a significantly higher rate of previous SARS-CoV-2 infections in patients who had refused vaccination (Group 5) at the final inclusion period.

Lastly, analyses were exploratory and not formally powered. The results therefore should be considered as hypothesis generating and interpreted with care. Nonetheless, as illustrated above, the BELCOMID results are in line with what was found in previous similar studies.

As a final remark, we want to highlight that even in a tertiary IMID patient population followed at two academic centers that played a major role in the regional management of the pandemic, there still remains a group of patients (Group 5) who refused every dose of SARS-CoV-2 vaccine up to the end of the study. This patient group experienced the highest rate of COVID-19 infections and had the lowest rates of S-antibody seroconversion. Vaccine hesitancy and low vaccine coverage in IMID patients in general has been a longstanding issue [41]. Qualitative studies have identified some key barriers to COVID-19 vaccine uptake in IMID patients such as the belief that vaccination could trigger IMID disease activity and concerns for safety with newly developed vaccine technology [21,71]. However, as illustrated above, these fears are mostly ungrounded. Incomplete vaccination or waning of immune response after vaccination may be of more importance than the risk or attenuating effect of certain IMID treatment modalities. Furthermore, as the COVID-19 “hype” tends to disappear from media channels, there remains an important task for IMID caregivers to keep their patients informed about the value and necessity of COVID-19 booster vaccines as long as SARS-CoV-2 remains endemic.

## 5. Conclusions

The BELCOMID study provides reassuring real-world evidence that counters concerns about severe COVID-19, reduced vaccination efficacy or increased vaccination adverse events in IMID patients.

No clear association between SARS-CoV-2 infection rate and IMID-treatment modality was found apart from a seemingly protective effect of anti-TNF treatment prior to vaccination. Infection rates increased over the course of the pandemic and were highest in patients that had refused every vaccine. Immune responses to vaccination may be blunted secondary to IMID-treatment modalities such as systemic steroids, rituximab, anti-TNF, JAKi and combined TIMT/IMM. Booster vaccination progressively increased immune responses and rates of IMID-flare up after COVID-19 disease or vaccination were low. All in all, the benefit of vaccination and repeated booster vaccination in IMID patients outweighs the potential risks. The monitoring of S-antibody concentration/titers may be considered to guide future booster vaccination.

## Figures and Tables

**Table 1:** Demographics per predefined vaccination group.

	Group 1	Group 2	Group 3	Group 4	Group 5
Number of patients	2144	1532	1283	147	40
Mean age yo (SD)	44.6 (35.5)	46.9 (30.8)	46.6 (32.7)	51.0 (15.7)	39.4 (14.4)
Age category					
<60 yo	1550 (72.3%)	1084 (70.8%)	930 (72.5%)	98 (66.7%)	35 (87.5%)
>/=60 yo	463 (21.6%)	417 (27.2%)	341 (26.6%)	49 (33.3%)	5 (12.5%)
Male/female	1088 (51.0%)/1047 (49.0%)	794 (51.9%)/737 (48.1%)	661 (51.6%)/621 (48.4%)	80 (54.4%)/67 (45.6%)	24 (60.0%)/16 (40%)
BMI in kg/m^2^ (mean (SD))	26.1 (4.95)	26.2 (5.06)	26.4 (4.72)	26.7 (5.14)	23.8 (3.63)
<18.5 kg/m^2^	54 (2.8%)	30 (2.3%)	20 (1.8%)	5 (4.1%)	1 (2.8%)
18–25 kg/m^2^	807 (42.0%)	553 (42.5%)	424 (38.9%)	41 (33.3%)	21 (58.3%)
25–30 kg/m^2^	691 (35.9%)	465 (35.7%)	429 (39.4%)	49 (39.8%)	11 (30.6%)
>30 kg/m^2^	371 (19.3%)	254 (19.5%)	217 (19.9%)	28 (22.8%)	3 (8.3%)
>25 kg/m^2^	1062 (49.5%)	750 (49.0%)	656 (51.1%)	82 (55.8%)	12 (32.5%)
Comorbidities					
Heart disease	200 (9.33%)	173 (11.3%)	120 (9.35%)	27 (18.4%)	1 (2.5%)
Chronic pulmonary disease (not asthma)	63 (2.94%)	57 (3.72%)	38 (2.96%)	7 (4.76%)	2 (5%)
Asthma	73 (3.4%)	51 (3.33%)	53 (4.13%)	8 (5.44%)	3 (7.50%)
CKD	49 (2.29%)	46 (3.00%)	41 (3.20%	4 (2.72%)	2 (5.00%)
Chronic liver disease	75 (3.50%)	90 (5.87%)	69 (5.38%)	7 (4.76%)	3 (7.50%)
Neurologic disease	44 (2.05%)	54 (3.52%)	47 (3.66%)	8 (5.44%)	0
Malignancy (history or active)	111 (5.18%)	83 (5.42%)	82 (6.39%)	2 (1.36%)	0
Hematologic disease	45 (2.10%)	45 (2.94%)	28 (2.18%)	5 (3.40%)	2 (5.00%)
HIV	2 (0.09%)	2 (0.13%)	2 (0.16%)	0	0
Diabetes mellitus	97 (4.52%)	78 (5.09%)	69 (5.38%)	7 (4.76%)	1 (2.50%)
No comorbidities	565 (26.4%)	388 (25.3%)	277 (21.6%)	42(28.6%)	9 (22.5%)
Active smoker	369 (17.2%)	261 (17.0%)	201 (15.7%)	24 (16.3%)	12 (30.0%)
Increased COVID-19 exposure risk *	1019 (47.5%)	621 (40.5%)	69 (49.8%)	80 (54.4%)	23 (57.5%)
IMID type					
Dermatologic	310 (14.5%)	239 (15.6%)	139 (10.8%)	42 (28.6%)	5 (12.5%)
HS	36 (12.2%)	21 (8.8%)	17 (12.4%)	2 (4.8%)	1 (20%)
Pso	226 (76.6%)	195 (81.6%)	100 (73.0%)	37 (88.1%)	4 (80.0%)
Atopic derm	33 (11.2%)	20 (14.6%)	20 (14.6%)	3 (7.1%)	0
Gastro/IBD	1336 (62.3%)	982 (64.1%)	920 (71.7%)	65 (44.2%)	31 (77.5%)
CD	838 (64.9%)	644 (66.3%)	589 (64.3%)	46 (71.9%)	19 (61.3%)
UC	404 (31.3%)	294 (30.2%)	295 (32.2%)	16 (25.0%)	12 (38.7%)
IPAA	37 (2.9%)	25 (2.6%)	25 (2.7%	1 (1.6%)	0
Undifferentiated colitis	13 (1.0%)	9 (0.9%)	7 (0.8%)	1 (1.6%)	0
Rheumatologic	498 (23.2%)	311 (20.3%)	224 (17.5%)	40 (27.2%)	4 (10.0%)
RA	256 (56.0%)	179 (60.1%)	127 (58.0%)	24 (61.5%)	2 (50.0%)
SpA	126 (27.6%)	61 (20.5%)	43 (19.6%)	5 (12.8%)	2 (50.0%)
PsoA	75 (16.4%)	58 (19.5%)	49 (22.4%)	10 (25.6%)	0
IMID-treatment modality					
TIMT	1580 (73.7%)	1232 (80.4%)	1073 (83.6%)	119 (81.0%)	38 (95.0%)
Infliximab	503 (23.5%)	394 (25.7%)	376 (29.3%)	23 (15.6%)	17 (42.5%)
Anti-TNF alpha	783 (36.5%)	594 (38.8%)	523 (40.8%)	60 (40.8%)	21 (52.5%)
Vedolizumab	328 (15.3%)	260 (17.0%)	270 (21.1%)	9 (6.1%)	7 (17.5%)
Rituximab	36 (1.7%)	23 (1.5%)	20 (1.6%)	1 (0.7%)	0
Anti-IL12/23/17 (grouped)	280 (13.1%)	228 (14.9%)	156 (12.2%)	40 (27.2%)	5 (12.5%)
Anti-IL12/23 (grouped)	199 (9.3%)	162 (10.6%)	119 (9.3%)	25 (17.0%)	3 (7.5%)
Anti-IL17	83 (3.9%)	66 (4.3%)	38 (3.0%)	15 (10.2%)	2 (5.0%)
Anti-IL23	69 (3.2%)	60 (3.9%)	29 (2.3%)	14 (9.5%)	1 (2.5%)
JAK-inhibitor	34 (1.6%)	36 (2.3%)	23 (1.8%)	1 (0.7%)	1 (0.7%)
IMM	456 (21.3%)	311 (20.3%)	225 (17.5%)	39 (26.5%)	8 (20.0%)
Combined TIMT + IMM	263 (12.3%)	197 (12.9%)	152 (11.8%)	24 (16.3%)	8 (20.0%)
N-IM (= non-TIMT/non-IMM at baseline)	113 (5.3%)	64 (4.2%)	44 (3.4%)	4 (2.7%)	1 (2.5%)
Received systemic steroids	229 (10.7%)	91 (5.94%)	59 (4.60%)	6 (4.08%)	4 (10.0%)
No active IMID disease ** during time period 1	1309 (61.05%)	1035 (67.56%)	951 (74.12%)	93 (63.27%)	31 (77.5%)

Abbreviations: yo: years old, SD: standard deviation, BMI: body mass index, CKD: chronic kidney disease, HIV: human immunodeficiency virus seropositivity, IMID: immune mediated inflammatory disease, HS: hidradenitis suppurativa, Pso: psoriasis, atopic derm(atitis), CD: Crohn’s disease, UC: ulcerative colitis, IPAA: ileal pouch anal anastomosis (post colectomy), RA: rheumatoid arthritis, SpA: spondyloarthritis, PsoA: psoriatic arthritis, TIMT: targeted immune-modulating treatment, IMM: immunomodulator, TNF: tumor necrosis factor, IL: interleukin, JAK: Janus kinase, PCR: polymerase chain reaction. * SARS-CoV-2 exposure risk was considered increased based on patients’ job description, recent travelling history or potential COVID-19 contact at healthcare facilities. ** Active disease: according to treating physician and/or based on patient reported outcome scores and/or requirement of steroid treatment and/or requirement of IMID-induced hospitalization.

**Table 2:** PCR positivity and seroconversion rates per vaccination group.

	Group 1	Group 2	Group 3	Group 4	Group 5
Number of patients	2144	1532	1283	147	40
Positive PCR over the past period	102 (5.1%)	87 (5.9%)	263 (20.5%)	42 (28.6%)	18 (45%)
N-antibody seroconversion	65/2108 (3.1%)	35/1481 (2.4%)	189/1240 (15.2%)	26/143 (18.2%)	9/39 (23.1%)
Ever had SARS-CoV-2 infection *	121 (5.7%)	131 (8.6%)	371 (28.9%)	57 (38.8%)	20 (50%)
S-antibody seroconversion	Not tested	1303/1370 (95.1%)	1216/1240 (98.1%)	143/143 (100%)	21/39 (53.8%)
S (−)/N (−)	N.A	66/1370 (4.8%)	24/1240 (1.9%)	0	16/39 (41.0%)
S (−)/N (+)	N.A	1/1370 (0.1%)	0	0	2/39 (5.1%)
S (+)/N (−)	N.A	1273/1370 (92.9%)	1027/1240 (82.8%)	117/143 (81.8%)	14/39 (35.9%)
S (+)/N (+)	N.A	30/1270 (2.2%)	189/1240 (15.2%)	26/143 (18.2%)	7/39 (17.9%)

Abbreviations: N: nucleocapsid protein, S: spike protein, (−): no seroconversion, (+) seroconversion, N.A: not applicable. * Based on N-seroconversion and/or PCR positivity.

**Table 3:** Associations between IMID treatment and SARS-CoV-2 response: PCR, antibody seroconversion and S-antibody concentration (exploratory analyses).

	Group 1	Group 2	Group 3	Group 4	Group 5
N	2144	1532	1283	147	40
Associations with positive SARS-CoV-2 PCR					
TIMT	OR 1.42 (95% CI 0.83–2.53, *p* = 0.22)	OR 0.97 (95% CI 0.49–2.06, *p* = 0.93)	OR 1.06 (95% CI 0.68–1.67, *p* = 0.81)	OR 0.625 (95% CI 0.14–2.66, *p* = 0.52)	Analysis not possible *
Infliximab	OR 1.10 (95% CI 0.65–1.78, *p* = 0.72)	OR 0.61 (95% CI 0.30–1.16, *p* = 0.15)	OR 1.14 (95% CI 0.80–1.63, *p* = 0.46)	OR 0.897 (95% CI 0.17–4.54, *p* = 0.89)	RR 1.56 (95% CI 0.75–3.21, *p* = 0.4125)
Anti-TNF	OR 1.17 (95% CI 0.75–1.81, *p* = 0.48)	OR 0.69 (95% CI 0.38–1.23, *p* = 0.22)	OR 1.11 (95% CI 0.80–1.55, *p* = 0.53)	OR 1.13 (95% CI 0.39–3.28, *p* = 0.81)	RR 1.83 (95% CI 0.78–4.33, *p* = 0.2571)
Rituximab	RR 1.70 (95% CI 0.47–6.09, *p* = 0.7619)	RR 1.56 (95% CI 0.25–9.91, *p* = 1)	RR 0.68 (95% CI 0.20–2.31, *p* = 0.7547)	Numbers too low for analysis	No rituximab patients
Anti-IL12/23/17 (combined)	OR 1.24 (95% CI 0.68–2.14, *p* = 0.47)	OR 2.02 (95% CI 0.999–3.86, *p* = 0.04)	OR 1.2 (95% CI 0.71–2.02, *p* = 0.48)	OR 1.10 (95% CI 0.32–3.74, *p* = 0.88)	RR 0.92 (95% CI 0.32–2.62, *p* = 1)
Anti-IL12/23	OR 1.55 (95% CI 0.83–2.73, *p* = 0.15)	OR 2.04 (95% CI 0.96–4.01, *p* = 0.049)	OR 1.22 (95% CI 0.68–2.16, *p* = 0.5)	RR 1.62 (95% CI 0.88–3.02, *p* = 0.3559)	RR 0.92 (95% CI 0.22–3.87, *p* = 1)
Anti-IL23	OR 2.51 (95% CI 0.95–5.95, *p* = 0.047)	OR 6.32 (95% CI 1.78–20.3, *p* < 0.01)	RR 1.98 (95% CI 1.25–3.13, *p* = 0.0592)	RR 1.92 (95% CI 1.01–3.63, *p* = 0.3708)	RR 1.92 (95% CI 1.32–2.80, *p* = 1)
Anti-IL17	RR 0.77 (95% CI 0.26–2.32, *p* = 0.84)	RR 1.65 (95% CI 0.56–4.88, *p* = 0.6166)	OR 1.11 (95% CI 0.32–3.47, *p* = 0.87)	RR 0.67 (95% CI 0.20–2.25, *p* = 0.7609)	RR 0.92 (95% CI 0.22–3.87, *p* = 1)
JAKi	RR 1.01 (95% CI 0.27–3.81, *p* = 1)	RR 0.88 (95% CI 0.13–5.93, *p* = 1)	OR 2.31 (95% CI 0.77–6.87, *p* = 0.13)	RR 2.48 (95% CI 1.87–3.29, *p* = 0.8554)	Numbers too low for analysis
Vedolizumab	OR 0.81 (95% CI 0.42–1.47, *p* = 0.51)	OR 0.90 (95% CI 0.41–1.78, *p* = 0.77)	OR 0.797 (95% CI 0.53–1.19, *p* = 0.28)	RR 0.59 (95% CI 0.11–3.32, *p* = 0.8805)	RR 1.28 (95% CI 0.52–3.11, *p* = 1)
IMM	OR 0.96 (95% CI 0.54–1.65, *p* = 0.9)	OR 0.93 (95% CI 0.39–1.98, *p* = 0.86)	OR 1.4 (95% CI 0.89–2.17, *p* = 0.14)	OR 1.45 (95% CI 0.36–6.04, *p* = 0.6)	RR 0.56 (95% CI 0.17–1.82, *p* = 0.495)
Combined IMM + TIMT	OR 1.29 (95% CI 0.68–2.31, *p* = 0.41)	RR 0.65 (95% CI 0.24–1.73, *p* = 0.5074)	OR 1.51 (95% CI 0.92–2.47, *p* = 0.1)	RR 0.97 (95% CI 0.43–2.19, *p* = 1)	RR 0.56 (95% CI 0.17–1.82, *p* = 0.495)
N-IM	OR 1.18 (95% CI 0.44–2.69, *p* = 0.71)	RR 0.90 (95% CI 0.29–2.76, *p* = 1)	OR 0.81 (95% CI 0.30–1.92, *p* = 0.64)	Numbers too low for analysis	Numbers too low for analysis
Systemic steroid use	OR 1.08 (95% CI 0.52–2.04, *p* = 0.83)	RR 0.59 (95% CI 0.15–2.33, *p* = 0.633)	OR 1.74 (95% CI 0.79–3.83, *p* = 0.16)	RR 0.80 (95% CI 0.16–4.08, *p* = 1)	RR 0.59 (95% CI 0.11–3.04, *p* = 0.887)
IFX vs. vedo	OR 1.42 (95% CI 0.70–3.00, *p* = 0.35)	OR 0.726 (95% CI 0.29–1.81, *p* = 0.48)	OR 1.24 (95% CI 0.77–2.00, *p* = 0.37)	RR 2.00 (95% CI 0.33–12.18, *p* = 0.7978)	RR 1.00 (95% CI 0.41–2.45, *p* = 1)
Anti-TNF vs. vedo	OR 1.34 (95% 0.70–2.73, *p* = 0.4)	OR 0.846 (95% CI 0.38–1.99, *p* = 0.69)	OR 1.24 (95% CI 0.79–1.96, *p* = 0.35)	RR 1.88 (95% CI 0.33–10.66, *p* = 0.7583)	RR 1.00 (95% CI 0.42–2.40, *p* = 1)
Anti-IL12/23/17 vs. vedo	OR 1.23 (95% CI 0.54–2.81, *p* = 0.63)	OR 2.08 (95% CI 0.84–5.26, *p* = 0.11)	OR 1.27 (95% CI 0.66–2.41, *p* = 0.47)	RR 1.87 (95% CI 0.31–11.09, *p* = 0.8337)	RR 0.75 (95% CI 0.21–2.66, *p* = 1)
Anti-TNF vs. anti-IL12/23/17	OR 0.90 (95% CI 0.49–1.74, *p* = 0.75)	OR 0.445 (95% CI 0.21–0.98, *p* = 0.04)	OR 0.89 (95% CI 0.51–1.57, *p* = 0.68)	OR 1.08 (95% CI 0.29–4.04, *p* = 0.91)	RR 1.33 (95% CI 0.47–3.78, *p* = 0.9755)
Anti-TNF vs. JAKi	RR 1.09 (95% CI 0.28–4.18, *p* = 1)	RR 0.97 (95% CI 0.14–6.73, *p* = 1)	OR 0.55 (95% CI 0.18–1.71, *p* = 0.29)	RR 0.47 (95% CI 0.33–067, *p* = 0.9769)	Numbers too low for analysis
Anti-TNF vs. rituximab	RR 0.65 (95% CI 0.18–2.39, *p* = 0.8711)	RR 0.556 (95% CI 0.09–3.65, *p* = 1)	RR 1.55 (95% CI 0.45–5.32, *p* = 0.6842)	Numbers too low for analysis	RR 1.26 (95% CI 0.61–2.59, *p* = 0.9171)
Associations with N-seroconversion					
TIMT	OR 1.29 (95% CI 0.68–2.68, *p* = 0.46)	OR 1.17 (95% CI 0.50–3.21, *p* = 0.73)	OR 0.82 (95% CI 0.54–1.28, *p* = 0.36)	RR 1.70 (95% CI 0.55–5.25, *p* = 0.4903)	Analysis not possible *
Infliximab	OR 0.69 (95% CI 0.33–1.32, *p* = 0.28)	OR 0.76 (95% CI 1.7–0.53, *p* = 0.53)	OR 1.05 (95% CI 0.73–1.50, *p* = 0.77)	OR 0.87 (95% CI 0.22–2.98, *p* = 0.83)	RR 1.62 (95% CI 0.51–5.12, *p* = 0.6584)
Anti-TNF	OR 0.55 (95% CI 0.29–0.98, *p* = 0.051)	OR 0.72 (95% CI 0.33–1.48, *p* = 0.38)	OR 1.03 (95% CI 0.74–1.44, *p* = 0.84)	OR 0.95 (95% CI 0.36–2.50, *p* = 0.92)	RR 1.07 (95% CI 0.34–3.40, *p* = 1)
Rituximab	RR 0.90 (95% CI 0.13–6.31, *p* = 1)	RR 2.15 (95% CI 0.31–14.94, *p* = 0.9677)	No rituximab patient had N-seroconversion	Numbers too low for analysis	No rituximab patients
Anti-IL12/23/17 (combined)	OR 2.00 (95% CI 0.99–3.76, *p* = 0.04)	OR 1.64 (95% CI 0.64–3.71, *p* = 0.26)	OR 0.86 (95% CI 0.49–1.42, *p* = 0.57)	OR 1.5 (95% CI 0.50–4.29, *p* = 0.46)	RR 1.94 (95% CI 0.55–6.85, *p* = 0.6939)
Anti-IL12/23	OR 2.85 (95% CI 1.41–5.38, *p* < 0.01)	OR 1.54 (95% CI 0.51–3.76, *p* = 0.39)	OR 0.79 (95% CI 0.42–1.41, *p* = 0.45)	RR 0.62 (95% CI 0.20–1.89, *p* = 0.5507)	RR 1.50 (95% CI 0.27–8.32, *p* = 1)
Anti-IL23	OR 4.54 (95% CI 1.60–11.10, *p* < 0.01)	RR 3.11 (95% CI 1.13–8.52, *p* = 0.0655)	OR 0.94 (95% CI 0.26–2.67, *p* = 0.92)	RR 0.37 (95% CI 0.05–2.52, *p* = 0.4456)	RR 4.75 (95% CI 2.57–8.79, *p* = 0.5174)
Anti-IL17	RR 0.77 (95% CI 0.19–3.11, *p* = 0.9694)	RR 1.32 (95% CI 0.32–5.38, *p* = 1)	OR 0.88 (95% CI 0.28–2.29, *p* = 0.8)	OR 2.41 (95% CI 0.44–11.9, *p* = 0.29)	RR 2.31 (95% CI 0.51–10.53, *p* = 0.9472)
JAKi	RR 0.98 (95% CI 0.14–6.87, *p* = 1)	RR 1.25 (95% CI 0.18–8.88, *p* = 1)	RR 1.20 (95% CI 0.49–2.93, *p* = 0.93)	No JAKi patient had N-seroconversion	RR 4.75 (95% CI 2.57–8.79, *p* = 0.5174)
Vedolizumab	OR 1.87 (95% CI 0.95–3.49, *p* = 0.056)	OR 0.837 (95% CI 0.28–2.07, *p* = 0.72)	OR 0.84 (95% CI 0.55–1.26, *p* = 0.41)	RR 0.60 (95% CI 0.09–3.91, *p* = 0.9031)	RR 0.57 (95% 0.09–3.96, *p* = 0.909)
IMM	OR 0.52 (95% CI 0.22–1.08, *p* = 0.1)	OR 1.64 (95% CI 0.72–3.57, *p* = 0.22)	OR 0.76 (95% CI 0.46–1.21, *p* = 0.26)	OR 1.11 (95% CI 0.31–3.73, *p* = 0.87)	RR 1.11 (95% CI 0.28–4.34, *p* = 1)
Combined IMM + TIMT	OR 0.72 (95% CI 0.27–1.59, *p* = 0.45)	OR 2.52 (95% CI 1.09–5.47, *p* = 0.023)	OR 0.83 (95% CI 0.46–1.42, *p* = 0.52)	OR 1.66 (95% CI 0.42–5.97, *p* = 0.45)	RR 1.11 (95% CI 0.28–4.34, *p* = 1)
N-IM	RR 1.16 (95% CI 0.43–3.13, *p* = 0.993)	RR 0.69 (95% CI 0.10–4.92, *p* = 1)	OR 1.39 (95% CI 0.54–3.13, *p* = 0.45)	RR 1.39 (95% CI 0.25–7.87, *p* = 1)	Numbers too low for analysis
Systemic steroid use	OR 0.60 (95% CI 0.17–1.58, *p* = 0.35)	RR 1.56 (95% CI 0.49–4.99, *p* = 0.7034)	OR 1.12 (95% CI 0.43–2.54, *p* = 0.81)	No syst steroid patient had N-seroconversion	RR 1.50 (95% CI 0.27–8.32, *p* = 1)
IFX vs. vedo	OR 0.48 (95% CI 0.20–1.08, *p* = 0.077)	OR 0.98 (95% CI 0.30–3.42, *p* = 0.97)	OR 1.2 (95% CI 0.75–1.94, *p* = 0.45)	RR 2.35 (95% CI 0.33–16.87, *p* = 0.6557)	RR 2.06 (95% CI 0.29–14.59, *p* = 0.7954)
Anti-TNF vs. vedo	OR 0.40 (95% CI 0.18–0.86, *p* = 0.019)	OR 0.90 (95% CI 0.31–2.96, *p* = 0.84)	OR 1.15 (95% CI 0.73–1.83, *p* = 0.56)	RR 1.80 (95% CI 0.26–12.23, *p* = 0.8581)	RR 1.67 (95% CI 0.23–11.94, *p* = 1)
Anti-IL12/23/17 vs. vedo	OR 1.09 (95% CI 0.46–2.54, *p* = 0.83)	OR 1.91 (95% CI 0.55–7.01, *p* = 0.31)	RR 1.04 (95% CI 0.64–1.70, *p* = 0.9804)	RR 1.80 (95% CI 0.26–12.64, *p* = 0.8841)	RR 2.80 (95% CI 0.34–23.06, *p* = 0.7353)
Anti-TNF vs. anti-IL12/23/17	OR 0.39 (95% 0.18–0.88, *p* = 0.02)	OR 0.55 (95% CI 0.20–1.6, *p* = 0.25)	OR 1.17 (95% CI 0.68–2.12, *p* = 0.59)	OR 0.64 (95% CI 0.19–2.12, *p* = 0.46)	RR 0.60 (95% CI 0.16–2.22, *p* = 0.863)
Anti-TNF vs. JAKi	RR 0.80 (95% CI 0.11–5.83, *p* = 1)	RR 0.65 (95% CI 0.09–4.87, *p* = 1)	RR 0.85 (95% CI 0.34–2.11, *p* = 0.9632)	Analysis not possible	RR 0.24 (95% CI 0.11–0.51, *p* = 0.6014)
Anti-TNF vs. rituximab	RR 0.88 (95% CI 0.12–6.38, *p* = 1)	RR 0.381 (95% CI 0.05–2.81, *p* = 0.8729)	Analysis not possible	Numbers too low for analysis	No rituximab patients
Associations with S-seroconversion					
TIMT		OR 0.28 (95% CI 0.10–0.65, *p* < 0.01)	RR 0.99 (95% CI 0.98–1.01, *p* = 0.8517)	S-antibody seroconversion in 100% of patients	Analysis not possible **
Infliximab		OR 0.96 (95% CI 0.53–1.83, *p* = 0.9)	OR 0.68 (95% CI 0.27–1.84, *p* = 0.41)		RR 1.42 (95% CI 0.80–2.53, *p* = 0.382)
Anti-TNF		OR 0.76 (95% CI 0.28–1.92, *p* = 0.57)	OR 1.03 (95% CI 0.43–2.65, *p* = 0.94)		OR 4.34 (0.80–30.8, *p* = 0.11)
Rituximab		OR 0.035 (95% CI 0.01–0.10, *p* < 0.001)	OR 0.037 (95% CI 0.01–0.13, *p* < 0.001)		No rituximab patients
Anti-IL12/23/17 (combined)		RR 1.04 (95% CI 1.01–1.06, *p* = 0.0408)	RR 1.02 (95% CI 1.01–1.03, *p* = 0.1268)		RR 0.34 (95% CI 0.06–2.01, *p* = 0.252)
Anti-IL12/23		RR 1.03 (95% CI 1.00–1.06, *p* = 0.2692)	RR 1.02 (95% CI 1.01–1.03, *p* = 0.2235)		RR 0.60 (95% CI 0.12–3.05, *p* = 0.8894)
Anti-IL23		RR 1.00 (95% CI 0.94–1.06, *p* = 1)	RR 1.02 (95% CI 1.01–1.03, *p* = 0.9334)		Analysis not possible (numbers too low)
Anti-IL17		RR 1.05 (95% CI 1.04–1.07, *p* = 0.1144)	RR 1.02 (95% CI 1.02–1.01, *p* = 0.7782)		Analysis not possible (numbers too low)
JAKi		RR 0.99 (95% CI 0.90–1.08, *p* = 1)	RR 0.97 (95% CI 0.89–1.07, *p* = 0.9078)		RR 1.90 (95% CI 1.41–2.57, *p* = 1)
Vedolizumab		OR 1.16 (95% CI 0.56–2.72, *p* = 0.71)	RR 1.02 (95% CI 1.01–1.03, *p* = 0.068)		RR 1.08 (95% CI 0.52–2.21, *p* = 1)
IMM		OR 0.28 (95% CI 0.16–0.49, *p* < 0.001)	OR 0.22 (95% CI 0.09–0.56, *p* < 0.01)		RR 0.91 (95% CI 0.43–1.96, *p* = 1)
Combined IMM + TIMT		OR 0.16 (95% CI 0.09–0.28, *p* < 0.001)	OR 0.15 (95% CI 0.06–0.38, *p* < 0.001)		RR 0.91 (95% CI 0.43–1.96, *p* = 1)
N-IM		RR 1.02 (95% CI 0.97–1.07, *p* = 0.7903)	RR 1.02 (95% CI 1.01–1.03, *p* = 0.6952)		Analysis not possible (numbers too low)
Systemic steroid use		OR 0.183 (95% CI 0.10–0.37, *p* < 0.001)	OR 0.06 (95% CI 0.02–0.19, *p* < 0.001)		RR 1.26 (95% CI 0.54–2.98, *p* = 1)
IFX vs. vedo		OR 0.76 (95% CI 0.28–1.92, *p* = 0.57)	RR 0.98 (95% CI 0.97–1.00, *p* = 0.1222)		RR 1.13 (95% CI 0.54–2.35, *p* = 1)
Anti-TNF vs. vedo		OR 0.64 (95% CI 0.25–1.48, *p* = 0.32)	RR 0.99 (95% CI 0.97–1.00, *p* = 0.1966)		RR 1.13 (95% CI 0.54–2.35, *p* = 1)
Anti-IL12/23/17 vs. vedo		RR 1.02 (95% CI 0.99–1.05, *p* = 0.4616)	RR 1.00 (95% CI 1.00–1.01, *p* = 1)		RR 0.35 (95% CI 0.054–2.264, *p* = 0.4884)
Anti-TNF vs. anti-IL12/23/17		RR 0.97 (95% CI 0.94–1.00, *p* = 0.1034)	RR 0.98 (95% CI 0.97–0.99, *p* = 0.2133)		RR 3.33 (95% CI 0.56–19.75, *p* = 0.1631)
Anti-TNF vs. JAKi		RR 1.02 (95% CI 0.93–1.11, *p* = 1)	RR 1.03 (95% CI 0.94–1.13, *p* = 0.8952)		RR 0.67 (95% CI 0.49–0.90, *p* = 1)
Anti-TNF vs. rituximab		OR 26.3 (95% CI 7.36–105, *p* < 0.001)	OR 27.5 (95% CI 5.55–131, *p* < 0.001)		No rituximab patients
Associations S-antibody concentration (log-transformed)					
TIMT		Mean ratio 0.65 (95% CI 0.50–0.84, *p* < 0.01)	Mean ratio 0.53 (95% CI 0.40–0.69, *p* < 0.001)	Number of observations too low for analysis	
Infliximab		Mean ratio 0.62 (95% CI 0.49–0.78, *p* < 0.001)	Mean ratio 0.48 (95% CI 0.39–0.59, *p* < 0.001)		
Anti-TNF		Mean ratio 0.57 (95% CI 0.46–0.70, *p* < 0.001)	Mean ratio 0.44 (95% CI 0.36–0.53, *p* < 0.001)		
Rituximab		Mean ratio 0.07 (95% CI 0.03–0.16, *p* < 0.001)	Mean ratio 0.06 (95% CI 0.03–0.13, *p* < 0.001)		
Anti-IL12/23/17 (combined)		Mean ratio 1.29 (95% CI 0.95–1.75, *p* = 0.1)	Mean ratio 1.24 (95% CI 0.91–1.68, *p* = 0.18)		
Anti-IL12/23		Mean ratio 1.32 (95% CI 0.93–1.86, *p* = 0.12)	Mean ratio 1.27 (95% CI 0.90–1.80, *p* = 0.17)		
Anti-IL23		Mean ratio 2.17 (95% CI 1.19–3.96, *p* = 0.011)	Mean ratio 2.1 (95% CI 1.02–4.33, *p* = 0.045)		
Anti-IL17		Mean ratio 1.23 (95% CI 0.70–2.19, *p* = 0.47)	Mean ratio 0.97 (95% CI 0.51–1.83, *p* = 0.92)		
JAKi		Mean ratio 0.65 (95% CI 0.33–1.27, *p* = 0.21)	Mean ratio 1.15 (95% CI 0.57–2.34, *p* = 0.7)		
Vedolizumab		Mean ratio 1.84 (95% CI 1.40–2.41, *p* < 0.001)	Mean ratio 2.05 (95% CI 1.62–2.59, *p* < 0.001)		
IMM		Mean ratio 0.59 (95% CI 0.45–0.78, *p* < 0.001)	Mean ratio 0.41 (95% CI 0.32–0.54, *p* < 0.001)		
Combined IMM + TIMT		Mean ratio 0.38 (95% CI 0.28–0.53, *p* < 0.001)	Mean ratio 0.31 (95% CI 0.23–0.43, *p* < 0.001)		
N-IM		Mean ratio 1.36 (95% CI 0.82–2.26, *p* = 0.23)	Mean ratio 2.2 (95% CI 1.25–3.85, *p* < 0.001)		
Systemic steroid use		Mean ratio 0.30 (95% CI 0.19–0.46, *p* < 0.001)	Mean ratio 0.32 (95% CI 0.19–0.53, *p* < 0.001)		
IFX vs. vedo		Mean ratio 0.43 (95% CI 0.31–0.59, *p* < 0.001)	Mean ratio 0.35 (95% CI 0.27–0.45, *p* < 0.001)		
Anti-TNF vs. vedo		Mean ratio 0.42 (95% CI 0.32–0.56, *p* < 0.001)	Mean ratio 0.34 (95% CI 0.26–0.43, *p* < 0.001)		
Anti-IL12/23/17 vs. vedo		Mean ratio 0.82 (95% CI 0.56–1.21, *p* = 0.32)	Mean ratio 0.66 (95% CI 0.49–0.90, *p* < 0.01)		
Anti-TNF vs. anti-IL12/23/17		Mean ratio 0.56 (95% CI 0.41–0.78, *p* < 0.001)	Mean ratio 0.53 (95% CI 0.38–0.72, *p* < 0.001)		
Anti-TNF vs. JAKi		Mean ratio 1.12 (95% CI 0.58–2.16, *p* = 0.74)	Mean ratio 0.55 (95% CI 0.26–1.14, *p* = 0.11)		
Anti-TNF vs. rituximab		Mean ratio 11.1 (95% CI 4.18–29.4, *p* < 0.001)	Mean ratio 10.5 (95% CI 4.02–27.3, *p* < 0.001)		
Associations with lowest S-antibody concentration quartile (Q) within group					
TIMT		OR 2.58 (95% CI 1.66–4.20, *p* < 0.001)	OR 2.54 (95% CI 1.57–4.32, *p* < 0.001)	RR 2.33 (95% CI 0.58–9.34, *p* = 0.3209)	Numbers too low for analysis (only 5 patients not in lowest S quartile)
Infliximab		OR 1.5 (95% CI 1.09–2.06, *p* = 0.012)	OR 2.8 (95% CI 2.07–3.79, *p* < 0.001)	OR 2.26 (95% CI 0.61–7.74, *p* = 0.2)	
Anti-TNF		OR 1.63 (95% CI 1.21–2.19, *p* < 0.01)	OR 3.52 (95% CI 2.61–4.79, *p* < 0.001)	OR 2.68 (95% CI 1.01–7.58, *p* = 0.052)	
Rituximab		OR 10.1 (95% CI 3.63–32.5, *p* < 0.001)	OR 7.89 (95% CI 2.83–25.40, *p* < 0.001)	Numbers too low for analysis	
Anti-IL12/23/17 (combined)		OR 0.80 (95% CI 0.50–1.25, *p* = 0.035)	OR 0.62 (95% CI 0.36–1.01, *p* = 0.068)	RR 0.25 (95% CI 0.06–1.00, *p* = 0.0461)	
Anti-IL12/23		OR 0.89 (95% CI 0.52–1.46, *p* = 0.65)	OR 0.47 (95% CI 0.23–0.87, *p* = 0.023)	No anti-IL12/23 patients in lowest Q	
Anti-IL23		OR 0.67 (95% CI 0.23–1.64, *p* = 0.43)	RR 0.31 (95% CI 0.08–1.19, *p* = 0.0824)	No anti-IL23 patients in lowest Q	
Anti-IL17		OR 0.54 (95% CI 0.19–1.28, *p* = 0.2)	OR 1.2 (95% CI 0.48–2.77, *p* = 0.68)	RR 0.81 (95% CI 0.21–3.13, *p* = 1)	
JAKi		OR 2.48 (95% CI 1.12–5.24, *p* = 0.019)	RR 0.41 (95% CI 0.11–1.56, *p* = 0.2326)	Numbers too low for analysis	
Vedolizumab		OR 0.68 (95% CI 0.44–1.02, *p* = 0.072)	OR 0.206 (95% CI 0.12–0.34, *p* < 0.001)	RR 2.23 (95% CI 0.81–6.12, *p* = 0.3239)	
IMM		OR 1.55 (95% CI 1.09–2.21, *p* < 0.015)	OR 2.42 (95% CI 1.68–3.47, *p* < 0.001)	OR 1.64 (95% CI 0.44–5.64, *p* = 0.44)	
Combined IMM + TIMT		OR 2.73 (95% CI 1.86–3.98, *p* < 0.001)	OR 3.12 (95% CI 2.08–4.66, *p* < 0.001)	OR 3.5 (95% CI 0.88–13.50, *p* = 0.066)	
N-IM		OR 0.82 (95% CI 0.35–1.68, *p* = 0.61)	RR 0.20 (95% CI 0.05–0.79, *p* < 0.01)	Numbers too low for analysis	
Systemic steroid use		OR 3.27 (95% CI 1.95–5.43, *p* < 0.001)	OR 2.07 (95% CI 1.07–3.90, *p* = 0.026)	RR 2.63 (95% CI 0.84–8.25, *p* = 0.3886)	
IFX vs. vedo		OR 1.99 (95% CI 1.25–3.26, *p* < 0.01)	OR 7.86 (95% CI 4.60–14.30, *p* < 0.001)	RR 0.78 (95% CI 0.25–2.48, *p* = 1)	
Anti-TNF vs. vedo		OR 1.95 (95% CI 1.25–3.12, *p* < 0.01)	OR 7.84 (95% CI 4.64–14.10, *p* < 0.001)	RR 0.75 (95% CI 0.27–2.09, *p* = 0.9014)	
Anti-IL12/23/17 vs. vedo		OR 1.01 (95% CI 0.54–1.88, *p* = 0.97)	OR 2.3 (95% CI 1.08–4.90, *p* = 0.03)	RR 0.15 (95% CI 0.03–0.77, *p* = 0.0539)	
Anti-TNF vs. anti-IL12/23/17		OR 1.75 (95% CI 1.08–2.93, *p* = 0.027)	OR 2.79 (95% CI 1.67–4.89, *p* < 0.001)	RR: 5.00 (95% CI 1.21–20.69, *p* = 0.0195)	
Anti-TNF vs. JAKi		OR 0.53 (95% CI 0.24–1.19, *p* = 0.11)	RR 3.75 (95% CI 0.99–14.12, *p* = 0.0274)	Numbers too low for analysis	
Anti-TNF vs. rituximab		OR 0.16 (95% CI 0.04–0.49, *p* < 0.01)	OR 0.24 (95% CI 0.07–0.76, *p* = 0.02)	Numbers too low for analysis	

Abbreviations: TIMT: targeted immune-modulating treatment, IMM: immunomodulator, N-IM: non-TIMT and non-IMM at baseline, IFX: infliximab, vedo: vedolizumab, JAKi: JAK-inhibitor. * no patients without TIMT with PCR positivity or N-seroconversion, therefore numbers too low for analysis. ** no patients without TIMT with N-seroconversion, therefore numbers too low for analysis. Results of multivariate analyses shown if the analysis sample consists of at least 5 patients in each subgroup, conditional treatment effects are estimated using binary logistic regression models adjusted for the propensity score of the respective treatment.

## Data Availability

The raw data supporting the conclusions of this article will be made available by the authors on reasonable request.
